# Bang Your Head: Using Heavy Metal Music to Promote Scientific Thinking in the Classroom

**DOI:** 10.3389/fpsyg.2016.00146

**Published:** 2016-02-10

**Authors:** Rodney M. Schmaltz

**Affiliations:** Department of Psychology, MacEwan UniversityEdmonton, AB, Canada

**Keywords:** scientific thinking, teaching resources, heavy metal, music, introductory psychology

While heavy metal music may not be something typically covered in an introductory psychology textbook, there are many useful resources from this area of popular culture that can help promote scientific thinking in the classroom. From hidden messages in Judas Priest's music to Slayer being accused of inciting murder, heavy metal music has a long history of unique instances that are directly related to psychology. By incorporating examples from the world of heavy metal, educators can discuss scientific thinking in a way that is engaging and memorable for students.

Helping students think like scientists—that is to apply the rigorous principles of hypothesis testing outside of the classroom—is a challenge (Willingham, 2008). Robert Cialdini proposed that creating mystery in the classroom is an effective means to engage students and promote learning (Cialdini, 2005). Specifically, Cialadini argued that instructors should frame a lecture in the same way a mystery writer frames a novel, by posing a puzzle and providing the information for the reader—or in this case, the student—to solve it. The question, or mystery, can be broadly stated as, “Can music lead people to commit harmful acts?”

Using the Cialadini approach of creating mystery, educators can frame a discussion around music as a way to introduce a variety of topics related to scientific thinking, such as logical fallacies, issues in research methodology, and biases in thinking. For example, the belief that there is a causal link between music and harm could be discussed in terms of the argumentum ad antiquitatem fallacy, also known as the appeal to traditional (e.g., Vaughn and Schick, 1999). For over two thousand years, there has been public concern about the impact of certain types of music on behavior. Aristotle stated that “…if over a long time (a person) habitually listens to music that rouses ignoble passions, his whole character will be shaped to an ignoble form” (Grout, 1988). As music has historically been associated with causing harm, people may fall prey to the argumentum ad antiquitatem fallacy and accept the claim of causality between music and harm, without examining any empirical evidence.

Further discussion of fallacies and biases can be grounded in cases where heavy metal has been implicated in graphic and disturbing crimes. Heavy metal music came under intense scrutiny in the 1980's when heavy metal artists, such as Judas Priest and Ozzy Osbourne, were blamed for adolescent violence and suicide (Martin et al., 1993; Weinstein, 2000) ^1^. The shocking nature of these crimes are memorable, and as such are easily brought to mind when people think of heavy metal music. By discussing the availability heuristic—basing the likelihood of an event on the ease with which it comes to mind—educators can challenge students to consider what evidence they have used to assess the impact of music on behavior (Kahneman et al., 1982).

To facilitate scientific thinking, especially in terms of methodological issues, educators can present cases in popular culture and challenge students to determine the validity of the claims made. One of the most famous cases of heavy metal being implicated with harm is of Judas Priest. The band was charged with planting a subliminal message in the song *Better By You, Better Than Me* (Moore, 1996; Bushong, 2002). Specifically, when the song is played backwards the phrase “Do It” can be heard^2^. In this case, two teenage boys who had spent several hours listening to Judas Priest while drinking alcohol and smoking marijuana went to a local park and attempted suicide with a shotgun. Judas Priest was eventually acquitted of any wrongdoing, though for a somewhat surprising reason. Rather than the case being dismissed on account of the clear empirical evidence that subliminal messages could not cause a person to commit suicide (e.g., Vokey and Read, 1985; Egermann et al., 2006; Moore, 2008), the band was found not guilty because the “Do It,” which can be heard backwards, was not *intentionally* placed in the song. This case can lead to an interesting class discussion on how extraordinary claims require extraordinary evidence. The claim that a backwards, subliminal message can lead someone to take their own life is an extraordinary claim. Students can be challenged to describe how they would experimentally test the impact of subliminal messages on behavior, followed by a class discussion of how the actual research was conducted in the field (e.g., Vokey and Read, 1985). This is an engaging example to help students better understand variable manipulation, demand characteristics, and issues of generalizability. At least in the case of subliminal messages, students will learn that music does not lead to problematic or harmful behavior^3^.

In terms of creating mystery in the classroom, Cialdini suggests that instructors need to “deepen the mystery” and provide more details to the “case.” While there is no evidence that subliminal messages in music produce changes in behavior, there are examples where the link between harm and music is less clear. Norwegian Black Metal, an extreme form of heavy metal music consisting of distorted guitars and vocals, has been associated with murder, arson, and even cannibalism (Moynihan and Soderlind, 2003). To highlight the alarming nature of some of the acts associated with this type of music, educators may want to provide examples incorporating bands such as Mayhem, whose lead singer committed suicide in the band's recording studio in 1991. Upon finding the body, rather than calling the police, the guitarist for the band took polaroid photos and collected pieces of the skull to make necklaces for those he deemed “worthy.”^4^

Another example of music associated with disturbing and harmful acts can be found in the case of the band Slayer. In 1996, two teenagers were charged in the murder of a 15-year-old girl (Horn, 2000). The boys claimed they took inspiration to commit the crime from lyrics in the Slayer songs *Postmortem* and *Dead Skin Mask*^5^. The parents of the victim sued Slayer and their record label for unlawfully marketing and distributing obscene and harmful products to minors (Phillips, 2001; Potter, 2003). The crimes committed in the name of Black Metal and Slayer are very disturbing. While reliance on the availability heuristic provides an explanation as to why people could overestimate the likelihood of music causing harm, the mystery is far from solved.

Students should be challenged with providing the nature of the claim, and then exploring the evidence supporting the claim (Bartz, 2002). Is the evidence sufficient to demonstrate a causal relationship between heavy metal and problematic and deviant behavior? One approach to further engage students is to divide the class into groups to act as the prosecutor or defense in a mock trial of the Slayer murder case. The value in using this case is that the real-world outcome is known. The case did not go to trial, as the perpetrators of the crime had a history of criminal behavior, drug and alcohol abuse, as well as other factors that clearly demonstrated that listening to the music of Slayer was not the cause of the horrific crimes^6^. Cases like Slayer and Mayhem can lead to fruitful class discussion regarding how correlation does not equal causation.

There is a correlational relationship, but not a causal one, between music preference and problematic behavior. People who engage in problematic or criminal behaviors are more likely to listen to problem music, such as Black Metal (e.g., Epstein et al., 1990; Hansen and Hansen, 1991); however, the style of music a person prefers does not allow us to predict any problematic behavior. Simply put, if someone is wearing a Mayhem t-shirt, we cannot make any predictions about the likelihood that this person will commit a criminal act. If we know though, that a person has burned down a church, we are able to predict which type of music they most likely prefer. In these cases, the impact of music on behavior is mediated by other variables such as psychoticism (North et al., 2005), sensation-seeking (Litle and Zuckerman, 1986; Arnett, 1992), or negative family relationships (Arnett, 1992; Took and Weiss, 1994).

One of the reasons heavy metal music ideally fits Cialdini's structure of creating mystery in the classroom is that many of the mysteries regarding heavy metal music and harm have been solved. An interesting example, and an ideal one for class discussion, is the impact of the Parent's Music Resource Centre (PRMC), formed in 1985 and led by Tipper Gore (Chastagner, 1999). The PMRC believed that the lyrics in heavy metal music were directly contributing to the rise in suicide attempts and sexual assault among adolescents (Sampar, 2005). The PMRC demanded that albums be censored, leading to the “Parental Advisory” sticker now found on many popular albums. The PMRC implemented measures specifically, putting warning labels on music and trying to ban certain types of music, in order to protect people from the supposedly harmful effects of listening to heavy metal music. The PMRC can be used as a way to introduce further logical fallacies, such as the emotional fallacy (e.g., Slovic and Peters, 2006) and the argument from authority (Smith, 2010). The evidence on which the PRMC based their decisions was entirely anecdotal, and the anecdotes were highly emotional. While the members of the PMRC portrayed themselves as experts, none of the members had sufficient expertise in understanding human behavior. The PMRC is an ideal discussion point, as research has been done to demonstrate that the claims made by the organization were incorrect.

Contrary to the concerns of the PMRC, people who were fans of heavy metal music in adolescence fared *better* in many aspects of their adult lives than people who were not fans. Howe et al. (2015) surveyed people who were adolescent fans of the heavy metal in the 1980's. In comparison to college students and to a middle-aged comparison group, heavy metal fans reported that they were happier during adolescence, and were better adjusted as adults^7^. While the Howe et al. (2015) study shows that listening to heavy metal does not appear to have any negative long-term effects, what about the impact of listening to aggressive music on people who are fans of heavy metal? The PMRC claimed that listening to problematic music would lead to a cause in aggression. Sharman and Dingle (2015) found that listening to extreme music actually led to an *increase* in positive emotions for people who enjoy this type of music. The data indicate that the PMRC would have been wise to direct their attention elsewhere.

Heavy metal is certainly not the only topic, let alone music, that is associated with problematic behavior. Instructors are encouraged to use Cialdini's approach of bringing mystery to the classroom with other elements of pop culture, such as film, videogames, comic books, and other forms of music to promote scientific thinking. The value of using examples in heavy metal is that instructors can refer to the research that sheds light directly on the relationship between harm and this style of music. By using examples from heavy metal music, instructors are able to pose the question of the relationship between harm and heavy metal, allow students to consider the claims, apply critical thinking skills, propose how these claims should be tested, and finally solve the mystery with data from the relevant literature.

## Author contributions

The author confirms being the sole contributor of this work and approved it for publication.

### Conflict of interest statement

The author declares that the research was conducted in the absence of any commercial or financial relationships that could be construed as a potential conflict of interest. The reviewer, MD, and handling Editor declared their shared affiliation, and the handling Editor states that the process nevertheless met the standards of a fair and objective review.

## Footnotes

^1^See North and Hargreaves (2008) for more historical examples of public panic regarding music.

^2^To enhance the impact of this example, I encourage educators to allow students to hear the audio of the backwards message in class. The backwards version of the song is available on YouTube.

^3^Judas Priest is arguably the most famous case of backwards messaging, though there are many more examples. A thorough list of songs including backwards messaging, which can be useful class material, can be found on Wikipedia (https://en.wikipedia.org/wiki/List_of_backmasked_messages). A personal favorite is “Weird Al” Yankovic's backwards message in the song, “I Remember Larry.” When played backwards, the listener hears, “Wow, you must have an awful lot of free time on your hands.”

^4^For further details on Black Metal and criminal behavior, see Moynihan and Soderlind (2003).

^5^To increase the impact of these examples, I recommend playing parts of these songs, or showing videos of the songs performed live. Both of these songs are available on YouTube.

^6^For a detailed example of how critical thinking skills can be related to a legal setting, see Ennis (1987).

^7^Howe et al. (2015) also surveyed groupies and heavy metal musicians. While not the focus of this paper, the results from groupies and musicians can also make for interesting class discussion.
